# Pectoralis minor index range of healthy 18–24-year-old students from a Kenyan public university

**DOI:** 10.4102/sajp.v81i1.2096

**Published:** 2025-02-28

**Authors:** Eugene C. Agweyu, Joseph M. Matheri, Benita Olivier, Elzette Korkie

**Affiliations:** 1 Department of Rehabilitation Sciences, School of Medicine, College of Health Sciences, Jomo Kenyatta University of Agriculture and Technology, Nairobi, Kenya; 2 Kenya Medical Training College, The Nairobi Hospital, Nairobi, Kenya; 3 Centre for Healthy Living Research, Oxford Institute of Allied Health Research, School of Sport, Nutrition and Allied Health Professions, Oxford Brookes University, Oxford, United Kingdom; 4 Wits Cricket Research Hub for Science, Medicine and Rehabilitation, Department of Physiotherapy, School of Therapeutic Sciences, Faculty of Health Sciences, University of the Witwatersrand, Johannesburg, South Africa; 5 Department of Physiotherapy, School of Health Care Sciences, Faculty of Health Sciences, University of Pretoria, Pretoria, South Africa

**Keywords:** pectoralis minor index, shoulder dysfunction, shoulder pain, muscle length, baseline PMI values

## Abstract

**Background:**

The pectoralis minor muscle (PMM) length is critical for shoulder movement and stability, often implicated in dysfunction and pain. The pectoralis minor index (PMI) quantifies this muscle’s length relative to body dimensions. Typical PMI values range from 10.0 cm to 12.5 cm in healthy adults, with data for Kenyan populations.

**Objectives:**

This study aimed to establish baseline PMI values among healthy 18–24-year-old university students in Kenya, examining variations by side dominance and sex to support clinical assessments.

**Method:**

A cross-sectional descriptive study recruited 289 healthy young adults from Jomo Kenyatta University of Agriculture and Technology (JKUAT) using stratified and simple random sampling. Data were collected through a self-developed, interviewer-administered questionnaire, achieving a 93.4% response rate. PMI values were measured in three postures: supine, relaxed, and standing.

**Results:**

In the standing relaxed position, the PMI mean was 10.6 cm on the dominant side and 11.2 cm on the non-dominant side, with significant variation indicated by a 95% confidence interval. A paired *t*-test revealed a significant difference between dominant and non-dominant sides (*p* < 0.0001).

**Conclusion:**

Baseline PMI values for Kenyan young adults show significant differences by dominance and sex. These findings provide a foundational reference for assessing PMI in clinical settings, supporting physiotherapists and clinicians in evaluating and treating shoulder dysfunction using precise muscle length data.

**Clinical implications:**

Establishing baseline PMI values assists physiotherapists in identifying deviations, enabling targeted interventions for shoulder dysfunction.

## Introduction

The pectoralis minor index (PMI) is a ratio used to assess the resting length of the pectoralis minor muscle (PMM) relative to the individual’s height (Sharma et al. 2020). It helps determine muscle tightness or shortening, which can contribute to shoulder dysfunction, pain and poor postural control. Pectoralis minor index originates from studies focusing on shoulder biomechanics. Measurement involves using callipers or other tools to quantify the distance between anatomical landmarks, such as the coracoid process and the fourth rib, then standardising it to the person’s height for comparison. It helps physiotherapists in assessing shoulder function. This study focuses on healthy 18–24-year-old students from a Kenyan public university. Most 18–24-year-old young adults worldwide are students in various institutions of higher learning and carrying backpacks filled with learning materials or personal effects is expected in this cohort (Luime et al. 2004). This practice can lead to poor posture and shoulder girdle pain, particularly in cases of heavier backpacks, and if wrong methods are used in carrying these backpacks (Sankaran et al. 2021). Poor posture, often attributed to emotional and muscular factors, can cause structural or positional changes if not corrected (Williams, Laudner & McLoda 2013). While postural changes typically occur in the spine, other body parts are also affected (Malepe et al. 2015). Research indicates that muscles can become lengthened or shortened when body segments are held out of alignment for extended periods (Zakeri et al. 2016), reducing their efficiency and increasing the risk of neuro-musculoskeletal pathologies.

The pectoralis minor is a fan-shaped muscle that is part of the shoulder girdle on the upper chest beneath the pectoralis major. The pectoralis minor and major muscles form part of the anterior wall of the axilla region (Lewis & Valentine 2007). The PMM originates from the third to fifth ribs, near their sternocostal junctions, and attaches to the coracoid process of the scapula. It is innervated by the medial and lateral pectoral nerves, branches of the brachial plexus (Finley et al. 2017). The PMM plays secondary and tertiary roles in scapula function, primarily stabilising it by drawing it antero-inferiorly against the thoracic wall (Bond 2016). During scapula protraction, the PMM pulls the scapula forward; during scapula depression, it pulls the scapula downward (Borstad 2006). During inspiration, the PMM also elevates the third through to the fifth ribs, expanding the chest cavity and lung capacity (Morais & Cruz 2016). Nijs et al. (2005) stress that the PMM is also a significant anatomical landmark as it divides the axillary artery into three parts and the axillary lymph nodes into three levels.

Clinicians often assess posture or alignment during their provision of physiotherapy services. This assessment determines the position of body segments about the standard frame or other bone segments. If malalignments are detected, physiotherapists can suggest corrective measures (Borstad 2006). Prolonged postural deviation can change soft tissue flexibility, joint congruency and muscle lengths. These changes may result in poor force distribution or abnormal joint movement, potentially causing pain, impaired motor function or performance and a decreased range of motion (Teopister 2018). Therefore, testing and measuring potential adaptations or changes is necessary to determine the most appropriate interventions (Cools et al. 2015).

The PMI is commonly used to assess potential postural adaptations. Different studies had reported PMI values, as assessed with a Vernier calliper, in apparently healthy participants (aged 20–40) ranging from 7.65 to 8.61 (8.1 ± 1 standard deviation [s.d.]) (Weber et al. 2016), young female tennis players ranging from 6.2 to 7.6 (6.9 ± 2 s.d.) and patients (mean age 46.2 years) diagnosed with shoulder impingement ranging from 6.8 to 11.4 (9.1 ± 2.3) (Luime et al. 2004). There was a need to verify these PMI values in the local context.

The lack of baseline data on the PMI range highlights a significant gap in both physiotherapy education and practice. Additionally, inconsistencies in previous studies regarding PMI values, measurement tools for assessing PMM length and participant positioning variations during evaluations have complicated the interpretation of these values (Heuchert, Kozieł & Spinek 2024). Discrepancies in the analytical methods employed to examine differences between sexes and to compare values between upper limbs further exacerbate this issue. These gaps underscore the urgent need for standardised data within the local context. Therefore, the present study addresses this need by investigating the research question: What is the normal PMI range among healthy students aged 18 to 24 years at a public university in Kenya?

## Research methods and design

### Study setting

The study was conducted at the Main Campus of Jomo Kenyatta University of Agriculture and Technology (JKUAT).

### Study population

The study targeted young adults aged 18–24 years studying at JKUAT. At the time of the study, the university had a student population of 20 000, with 60% attending the main campus.

### Sampling and sampling procedure

A sample size of 289 healthy 18–24-year-old young adults, determined using the Cochran (1977) formula, was recruited.

The Cochran formula used in this study is described in Equation 1:


$$n_{0}=\frac{Z^{2}p(1-p)}{e^{2}},$$ [Eqn 1]

where *n*_0_ is the required sample size, *Z* is the *Z*-score (standard score corresponding to the desired confidence level, for example, 1.96 for 95% confidence), *p* is the estimated proportion of the population (often set at 0.5 if unknown, for maximum variability), and *e* is the margin of error (desired level of precision, such as 0.05 for 5%).

Both stratified and simple random sampling techniques were employed to select the study sample. A stratified sampling technique was used to categorise the study participants based on the year of study. A simple random sampling technique was used to select individual participants from each stratum, eliminating representative bias as it gave each population item an equal chance of being sampled. The researchers then evaluated the participants’ dominant shoulder, the shoulder typically used to carry their learning materials, and whether they used a backpack or a handbag.

### Instrumentation and outcome measures

**FIGURE 1:**
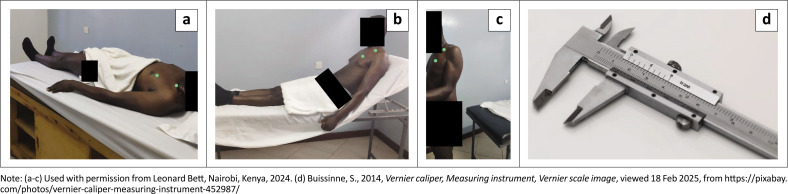
Participants’ pectoralis minor index (PMI) values were measured in three postures, (a) supine, (b) relaxed, and (c) standing using (d) a vernier caliper. The green dots indicate the lower (origin) and upper (insertion) surface markings for the measurements.

An interviewer-administered questionnaire (which sampled 289 participants from the institution) was used as the study tool and contained questions guided by the research objectives. A Vernier calliper was used to perform the PMM length measurements. The reliability of the Vernier calliper was Intraclass correlation coefficient (ICC) of 0.82–0.87 (Borstad & Ludewig 2005). The primary outcome measures were the participants’ PMI values in the three postures – supine, relaxed and standing (see Figure 1).

### Procedure

The researcher began by explaining the study’s purpose to potential participants, requesting their voluntary participation. Those who agreed were asked to provide written consent before their involvement proceeded. Upon obtaining consent, the researcher conducted an upper quadrant assessment to rule out any shoulder pathology or pain among participants.

Eligible participants were positioned supine, and vital anatomical landmarks were identified and marked with a dark marker. Prior studies with young, healthy adults indicate that PMI (Pectoralis et al.) values typically range between 6.5% and 8% of either body height or shoulder width. However, these values may vary depending on sample demographics (Moffatt, Simmons & Lynch-Aird 2016). Pectoralis minor muscle (Pectoralis et al.) length was measured with participants standing in a relaxed posture, allowing the scapula to retract fully without movement and again in a supine position.

The participants were reassessed in their relaxed, natural standing posture and the necessary changes to the location of the landmarks. The landmarks were the fourth rib (that is, the superior aspect of the fourth rib just lateral to the sternum) and the coracoid process (that is, the anteromedial aspect of the coracoid process) (Rosa et al. 2017). The origin and insertion of the PMM were marked with a skin marker in the supine (Borstad 2008). Participants were positioned supine, hands on the abdomen and scapulae retracted before measuring bilateral PMM length using a Vernier calliper (Ha et al. 2012). Marking of the origin and insertion was proven to have construct validity and had an ICC of 0.96 in the study by Borstad and Ludewig (2005). Positioning of the participant with elbow flexion and scapulae retraction followed biomechanical principles of muscle length evaluation (Dos Santos et al. 2021). The researcher used a Vernier calliper. Two examiners conducted the PMM length measurements. To minimise bias from the rater, the palm was placed with the metre facing away from the rater during all the measurements.

A second examiner read the dial on the calliper, recorded the measured value *t* and returned the calliper to position zero. Pectoralis minor was measured three times in relaxed, actively lengthened and passively lengthened positions, respectively (three times each), but the order was random. The researcher retraced and re-marked the landmarks on the body for measures done in lengthened conditions. In the meantime, one examiner stabilised the participant’s trunk by pushing the participant’s shoulder in a posterior/superior direction using the distal end of the humerus until a firm tissue resistance was obtained. In supine, the hand rested on the abdomen to eliminate biceps brachii insufficiency. The PMI for each participant was obtained by dividing the resting muscle’s measurement length by the participant’s height and multiplying the quotient by 100. This measurement approach was based on a cadaveric study by Muraki and others (Hasan et al. 2023) that determined the suitable stretching methods for the PMM.

### Data management

Data entry was done twice into the Statistical Package for Social Science (SPSS version 25.0), and descriptive statistics were done and compared between the two data sets to ensure correctness (Abu-Bader 2021). Once the correct data entry was completed, an analysis was performed using SPSS. The study data were analysed using descriptive statistics: percentages, frequencies, mean and standard deviation. Further, the independent samples *t*-test was used to compare the PMI mean values between males and females, while the paired *t*-test was used to compare the PMI mean values between the dominant and non-dominant sides. Both tests were done at 95% confidence level. Two diagnostic tests, normality and homoscedasticity tests, were also performed before the independent and paired *t*-tests were performed. The results were presented in tables.

### Ethical considerations

The National Commission for Science, Technology & Innovation (NACOSTI) obtained the study’s authority. Ethical approval was sought from the JKUAT Institutional Ethics Review Committee (IERC). The researcher provided a written information sheet before seeking the participant’s consent. The researcher informed the participants that being part of the study was voluntary and that no one would be penalised if they chose to withdraw themselves at any data collection stage. Participants were informed of the study’s purpose and requested their consent before being allowed into the study. They were also assured of the confidentiality of whatever information was gained from them, that the information would only be used for the study and that any emerging issues would only be cited anonymously.

## Results

### Demographic characteristics of the respondents

A response rate of 89.5% (*N* = 289) was achieved. The researchers assessed the respondents’ gender, age, education programme and academic year, with the results presented in Table 1.

**TABLE 1:** Respondents’ demographic characteristics.

Variable	Category	*n*	%
Gender	Male	171	59.2
	Female	118	40.8
	Total	289	100.0
Age (years)	18–20	122	42.2
	21–24	167	57.8
	Total	289	100.0
Education programme	Certificate	27	9.3
	Diploma	96	33.2
	Undergraduate	156	54.0
	Postgraduate	10	3.5
	Total	289	100.0
Academic year	1	39	13.5
	2	128	44.3
	3	92	31.8
	4	30	10.4
	Total	289	100.0

The researchers also evaluated the respondents’ height and weight measurements. The mean height for the male respondents was mean ± s.d. = 169.4 cm, with most male respondents (66.7%) having a height range of mean ± s.d. = 168 cm – 172 cm. Similarly, the mean height for the female respondents was 159.1 cm, with most female respondents (72.9%) having a height range of mean ± s.d. = 157 cm – 161 cm. The mean weight for the male respondents was 63.8 kg, with most male respondents (70.2%) having a weight range of 60 kg – 66 kg. Similarly, the mean weight for the female respondents was 61.6 kg, with most female respondents (77.1%) having a weight range of 58 kg – 64 kg.

### Dominant shoulder of the respondents

The results in Table 2 indicate that 242 (83%) of the respondents used a backpack to carry their learning materials. The dominant side for carrying these materials was the right shoulder, as 229 (79.2%) respondents reported.

**TABLE 2:** Tests of normality.

Rating categories	PMI values†		
Position	Statistic	*df*	Sig.
**Posture: Supine lying**			
Relaxed	1.00	289	0.77§
Passively lengthened	0.99	289	0.27‡
Actively lengthened	1.00	289	0.59§
**Posture: Resting**			
Relaxed	1.00	289	0.74§
Passively lengthened	0.99	289	0.30‡
Actively lengthened	1.00	289	0.90§
**Posture: Standing**			
Relaxed	0.99	289	0.51‡
Passively lengthened	0.99	289	0.11‡
Actively lengthened	0.99	289	0.23‡

*df*, degrees of freedom; PMI, pectoralis minor index; Sig., significance.

† , Shapiro-Wilk test;

‡ , Lilliefors Significance Correction;

§ , This is a lower bound of the true significance.

### Inferential statistics

#### Diagnostic test results

The normality test results are shown in Table 2.

Shapiro-Wilk tests revealed that there was a significant difference (*p* < 0.05) in PMI values among the different postures (supine lying, resting and standing) and at the three different positions (relaxed, passively lengthened and actively lengthened), respectively (Table 2). Table 3 presents the homoscedasticity test results based on Levene’s test for equality of variances.

**TABLE 3:** Tests of homogeneity of variance.

Rating categories		Levene statistic	*df*1	*df*2	*p*
SLR	Based on mean	2.69	1	287	0.10
SLPL	Based on mean	5.19	1	287	0.02
SEAL	Based on mean	0.70	1	287	0.40
RR	Based on mean	1.96	1	287	0.16
RPL	Based on mean	14.78	1	287	0.00
RAL	Based on mean	0.32	1	287	0.57
SR	Based on mean	5.79	1	287	0.02
SPL	Based on mean	2.04	1	287	0.15
SAL	Based on mean	1.17	1	287	0.28

SLR, supine lying relaxed position; SLPL, supine lying passively lengthened position; SEAL, supine extended actively lengthened position; RR, resting relaxed position; RPL, resting passively lengthened position; RAL, resting actively lengthened position; SR, standing relaxed position; SPL, standing passively lengthened position; SAL, standing actively lengthened position; *df*, degrees of freedom.

The homogeneity of variance test results shown in Table 4 indicates that the Levene test’s significance values for supine lying relaxed position – SLR (*p* = 0.102), supine lying actively lengthened position – SLAL (*p* = 0.405), resting relaxed position – RR (*p* = 0.162), resting actively lengthened position – RAL (*p* = 0.572), standing passively lengthened position – SPL (*p* = 0.154) and standing actively lengthened position – SAL (*p* = 0.280) were all greater than the set alpha value of 0.05.

**TABLE 4:** Group descriptive statistics (*N* = 289).

Rating categories	Gender	*n*	Mean	s.d.	s.e.	Mean difference	
						Male - Female	95% CI
SLR	-	-	-	-	-	0.1	0.02, 0.30
	Male	171	9.92	0.63	0.05	-	-
	Female	118	9.76	0.71	0.07	-	-
SLPL	-	-	-	-	-	0.27	0.10, 0.44
	Male	171	10.59	0.75	0.05	-	-
	Female	118	10.31	0.90	0.08	-	-
SLAL	-	-	-	-	-	0.05	−0.07, 0.19
	Male	171	10.13	0.67	0.05	-	-
	Female	118	10.07	0.61	0.05	-	-

Note: Mean, s.d., s.e., mean difference and 95% CI is given in cm.

CI, confidence interval; CM, centimetre; s.d., standard deviation; s.e., standard error; SLR, supine lying relaxed position; SLPL, supine lying passively lengthened position; SLAL, supine lying actively lengthened position.

### Paired *t*-test results

The researchers sought to determine whether there was a difference between the range of PMI on the dominant and non-dominant sides of healthy 18–24-year-old young adults. To achieve this objective, we performed a paired *t*-test of the mean PMI values of the respondents’ dominant and non-dominant sides while in a standing relaxed position. The results indicate that the mean PMI for the non-dominant sides of the respondents was 11.22 with a standard deviation of 1.051, while that of their dominant side was 10.6 with a standard deviation of 1.002. This denotes a difference in PMI range between the dominant and non-dominant sides of the healthy 18–24-year young adults. The paired samples *t*-test yielded a *t*-value of −7.301 and a significance value of *p* = 0.000. Given that the paired samples *t*-test significance value of 0.000 was less than the set alpha value of 0.05, the study’s null hypothesis was rejected, and its alternate hypothesis was accepted. We thus concluded that the difference between the range of PMI on the dominant and non-dominant sides of the healthy 18–24-year-old young adults was statistically significant.

### Supine lying posture findings

The findings on the group’s descriptive statistics for the independent samples *t*-test for the supine lying posture are presented in Table 4.

Results in Table 4 indicate a difference in PMI range values between males and females aged 18–24 years, with the male respondents having higher mean PMI values than their female counterparts in the three positions of supine lying posture.

## Discussion

The current study provides valuable information about the physical characteristics and variability in PMI among young adults in Kenya. The sample population (*N* = 289) included both male (59.2%) and female (40.8%) students, offering a gender-diverse perspective on PMI values. The age distribution leaned slightly towards older students (21–24 years, 57.8%) compared to the 18–20 age group, representing a balanced spread within the typical university age range. This age spread is particularly relevant, as muscle development, flexibility and postural characteristics may vary with age, even within this relatively young population.

This study also reveals that PMI values across various postures and positions largely follow a normal distribution. Each posture (supine lying, resting and standing) was assessed in three muscle states: relaxed, passively lengthened and actively lengthened. In all cases, the *p*-values are above the joint significance threshold of 0.05, with relaxed, passively lengthened and actively lengthened PMI values showing non-significant results (*p* > 0.05) across all positions. The normality of PMI values across all positions and postures suggests that the data collected in this study are symmetrically distributed and appropriate for further parametric analysis. This finding supports the reliability of the data in assessing PMI variations in a healthy young adult population. It provides a solid basis for examining factors influencing PMI in different postural settings. A similar study by Carvalho et al. (2019) supports this finding.

The current study found that the dominant shoulder among the respondents was the right shoulder, which was the one used by most 18–24-year-olds while carrying their learning materials. This agreed with the findings of several other studies (Sharma et al. 2020), which also reported the right shoulder as the dominant shoulder among most of their study participants. Similar observations were also made by Cools et al. (2015), Lewis and Valentine (2007), and Sharma et al. (2020), with most of the study participants establishing their right shoulder as their dominant side.

This study’s finding revealed that the participants’ dominant side had significantly lower PMI values when compared with the non-dominant side, suggesting that the dominant side is more susceptible to having a shorter PMM length. This has been attributed to the fact that the dominant side is more frequently used by the participants (Kendall et al. 2005). Consequently, the muscles can become lengthened or shortened, mainly when body segments are held out of alignment for extended periods (Zakeri et al. 2016). This may, however, lead to a reduction in the efficiency of PM muscle; this may subsequently increase the risk of neuro-musculoskeletal pathology. This finding is in agreement with the findings of other studies: (Stokes et al. 2016) and (Reina-Ruiz et al. 2023). This agreed with the findings of Komati, Korkie and Becker (2020), who, in a cross-sectional, observational quantitative study among healthy student participants drawn from a South African university, reported that the participants’ dominant side had statistically significantly lower PMI values than their non-dominant side. Similarly, Sharma et al. (2020), in an empirical evaluation of PMI values in an Indian population, also reported that differences existed in the participants’ PMI values between their dominant and non-dominant sides, and the difference was statistically significant. They established that PMI values on the participants’ dominant side were lower compared to their non-dominant side PMI values (Sharma et al. 2020). Similar results, on the dominant side having significantly lower PMI values than the non-dominant side, were also reported by Cools et al. (2015) and Struyf et al. (2014). Researchers attribute this finding to the fact that the dominant side is more robust and most frequently used; therefore, it is more susceptible to having a shorter PM muscle length than the less frequently used non-dominant side, as argued by Kendall et al. (2005).

The researchers also performed the independent samples *t*-test of the mean PMI values of the respondents, based on their gender, while in relaxed, passively lengthened and actively lengthened positions of three different postures – supine lying, resting and standing. This was done to determine whether there was a difference in PMI range values between healthy males and females who took part.

On the supine lying relaxed position, the mean PMI range values yielded an independent sample *t*-value of 2.107 and a significance value of 0.036. Consequently, we concluded that the difference in PMI range values between males and females aged 18–24 years young adults in the supine lying relaxed position was statistically significant. For the supine lying passively lengthened position, the mean PMI range values yielded an independent sample *t*-value of 2.727 and a significance value of 0.007. Consequently, we concluded that the difference in PMI range values between males and females aged 18–24 years young adults in the supine lying passively lengthened position was statistically significant. In the supine lying actively lengthened position, the mean PMI range values yielded an independent sample *t*-value of 0.731 and a significance value of 0.465. This result indicates no statistically significant difference between the PMI values of male and female participants in this position, as the *p*-value is considerably higher than the conventional alpha level of 0.05. This suggests that the differences observed in PMI between genders in the actively lengthened supine position may be because of random variation rather than any underlying physiological factors. A similar study by Jameson (2020) found significant differences in PMI values between genders in various positions, highlighting that males generally exhibit larger PMI values because of greater muscle mass and shoulder mechanics.

These findings corroborate Neophytou, Aginsky and Benjamin (2017), who also reported significant differences in PMI values among their study participants in supine lying, relaxed posture and passively lengthened posture (as reported in the current study). However, in contrast to the current study, Eraslan et al. (2021) reported significant differences in PMI values among their participants in supine lying actively lengthened posture. At the same time, the current study found the difference in PMI values among its participants as not statistically significant. Lee, Im and Kim (2020) and Bond (2016) attributed the significant differences to the role of the lower Trapezius on the PMM length and the scapula. Similar observations as to the existing significant differences in PMI range values between male and female healthy participants on the three supine lying postures (supine lying relaxed position, supine lying passively lengthened position and supine lying actively lengthened position) were reported by Struyf et al. (2014) and Rosa et al. (2017). The current study attributes these findings to sustained postural position changes and active use of the upper extremities.

In the resting relaxed position, the mean PMI range values yielded an independent sample *t*-value of 0.882 and a significance value of 0.378. The *t*-value of 0.882 and a significance (*p*) value of 0.378 indicate that the difference in PMI between the groups tested is not statistically significant at the conventional alpha level of 0.05. This means that any observed difference in PMI range values is likely because of chance rather than a meaningful effect. Thus, the PMI range values for the groups in this study do not significantly differ. This finding is consistent with those done by Pittner et al. (2020) and Reker (2005). In the resting passively lengthened position, the mean PMI range values yielded an independent sample *t*-value of 2.261 and a significance value of 0.025. This indicates a statistically significant difference in the PMI range values between the compared groups, as the *p*-value is less than the conventional threshold of 0.05. This suggests that the observed difference is unlikely because of random chance, implying that factors influencing the PMI differ significantly between the groups studied. A similar study by Bond (2016) highlighted a significant correlation between shoulder mobility and PMI, suggesting that increased lengthening of the pectoralis minor may benefit shoulder function and posture. Inoue et al. (2020) concluded that lengthening exercises could reduce the tightness of the pectoralis minor, supporting the current study’s findings regarding the importance of shoulder positioning on PMI. In contrast, Rasmussen et al. (2021) did not find statistically significant differences in PMI values across various shoulder positions in their study of a sedentary population.

Consequently, this study concluded that the difference in PMI range values between males and females aged 18–24 years young adults in the resting actively lengthened position was statistically significant (Komati et al. 2020). Similar observations were made that the differences in PMI values between male and female student participants drawn from a South African university were statistically significant in the resting passively lengthened and actively lengthened positions (Komati et al. 2020). Similar findings were also reported by Ebaugh et al. (2018). Pectoralis minor index range values significantly differed among the study participants in the resting posture’s two positions (passively and actively lengthened). Significant differences in PMI values among study participants in passively and actively lengthened positions of resting posture were also reported in studies by Rosa et al. (2017). The difference in PMI values among study participants in resting relaxed positions was also reported as not statistically significant in the findings by Komati et al. (2020). However, Struyf et al. (2014) and Rosa et al. (2017) found the difference statistically significant, which could be attributable to the fact that the inclusion criteria varied from the one used in our study.

On the standing relaxed position, the mean PMI range values yielded an independent sample *t*-value of 1.461 and a significance value of 0.145. Consequently, we concluded that the difference in PMI range values between males and females aged 18–24 years young adults in the standing relaxed position was not statistically significant. For the standing passively lengthened position, the mean PMI range values yielded an independent sample *t*-value of 2.603 and a significance value of 0.010. On the standing actively lengthened position, the mean PMI range values yielded an independent sample *t*-value of 1.084 and a significance value of 0.279. Consequently, we concluded that the difference in PMI range values between males and females aged 18–24 years studying at JKUAT in the standing actively lengthened position was not statistically significant. Studies by Struyf et al. (2014) and Rosa et al. (2016) also found the difference in PMI values in the standing passively lengthened position to be statistically significant among their study participants, observations also shared by Malepe et al. (2015) and Ebaugh et al. (2018) in their studies. The current study’s findings on statistical non-significance of differences in PMI values in standing relaxed position among study participants were also reported in studies by Lewis and Valentine (2007). However, the difference in PMI values among study participants in the actively lengthened position of the standing posture was reported to be statistically significant in findings by Malepe et al. (2015) and Borstad (2006), which was in contrast to the current study’s findings.

## Conclusion

This study offers valuable insights into the PMI variability among healthy young adults in Kenya, focusing on a diverse sample of 289 university students. The findings indicate that PMI values across different postures and muscle states predominantly follow a normal distribution, providing a reliable basis for further parametric analyses. Notably, gender differences were observed in several postures, with significant variations in the relaxed and passively lengthened positions, while no significant differences were found in the actively lengthened supine position. This suggests that the influence of gender on PMI may vary depending on specific postural and muscle states. The dominance of the right shoulder among participants highlighted potential muscle imbalances, as the dominant side exhibited significantly lower PMI values than the non-dominant side. This finding implies that habitual use of the dominant side may lead to muscle length and function adaptations, potentially increasing the risk of musculoskeletal disorders over time. The implications of these results extend beyond mere academic curiosity; they emphasise the necessity of considering gender, posture and shoulder dominance when evaluating PMI in young adults. The study’s results align with existing literature, confirming the importance of understanding how factors such as posture and activity level influence PMI. This research paves the way for future studies exploring muscle dynamics and postural alignment in various populations by establishing a baseline for PMI values in a Kenyan university setting. The findings of this study significantly contribute to understanding the factors influencing PMI among young adults. Continued exploration of these relationships can enhance our knowledge of musculoskeletal health, inform strategies for improving physical function and prevent musculoskeletal disorders, ultimately benefiting the health and well-being of young adults in Kenya and beyond.
